# Molecular mechanism of ziresovir targeting the fusion glycoprotein of respiratory syncytial virus

**DOI:** 10.1371/journal.ppat.1013864

**Published:** 2026-01-23

**Authors:** Mengrong Yan, Jingjing Zou, Zhao Gao, Haiqing Yuan, Jim Zhen Wu, Gang Zou, Fengjiang Liu, Wei Peng

**Affiliations:** 1 Innovative Center for Pathogen Research, Guangzhou National Laboratory, Guangzhou, China; 2 Institute of Pathogenic Biology, Hengyang Medical College, Hunan Provincial Key Laboratory for Special Pathogens Prevention and Control, University of South China, Hengyang, China; 3 Shanghai Ark Biopharmaceutical Co., Ltd, Shanghai, China; 4 State Key Laboratory of Respiratory Disease, the First Affiliated Hospital of Guangzhou Medical University, Guangzhou Medical University, Guangzhou, China; Purdue University, UNITED STATES OF AMERICA

## Abstract

Respiratory syncytial virus (RSV) is a leading cause of lower respiratory tract infections in infants and the elderly worldwide. Although prophylactic monoclonal antibodies and RSV vaccines are available for preventing severe RSV infection, unmet medical need remains for an effective antiviral agent to treat patients who do not benefit from these interventions. Ziresovir (formerly AK0529) is a potent, selective, and orally bioavailable RSV fusion inhibitor with proved antiviral efficacy and clinical benefits. To understand the molecular mechanism of action, we computationally modeled ziresovir with the RSV fusion (F) protein. Here, we present a cryo-EM structure of the RSV F protein-ziresovir complex, elucidating the molecular interactions underlying the drug binding, revealing ziresovir specifically binds to the central cavity within the metastable prefusion conformation of RSV F protein. Leveraging this structural insight, we engineered site-directed RSV mutants guided by both the cryo-EM binding model and drug-resistant RSV variants for fusion inhibitors identified *in vitro*, and demonstrated that these resistant viruses do not replicate as efficient as wild-type RSV and indicated a fitness cost for viral escape from drug treatment. Collectively, these findings unveil the structural mechanism of ziresovir-mediated viral inhibition, providing a framework for developing the next-generation RSV fusion inhibitors.

## Author summary

RSV is a common respiratory virus and can cause serious lung infections, especially in infants, older adults, and people with serious medical problems. Preventive strategies, including prophylactic monoclonal antibodies and RSV vaccines, have been developed to mitigate severe RSV infections, a critical unmet medical need persists. There are no orally administered antiviral agents for RSV infection that are clinically available globally. Ziresovir which is a potent, selective, and orally bioavailable RSV fusion inhibitor has been proven antiviral activity and confirmed clinical benefits. In this study, we test antiviral activity of ziresovir against RSV clinical isolates, which suggests that ziresovir have broadly activity against clinical isolates. Although, we assessed viral fitness for six ziresovir treatment-emergent F subsitutions and test susceptibility to ziresovir of these ziresovir treatment-emergent F substitutions contained recombinant RSV variants. To elude the structure basis for these F substitutions against ziresovir treatment, we determined the cryo-EM structure of the complex formed by the RSV F trimer protein and ziresovir that performed to explicitly elucidate the binding mode of ziresovir to F protein. We further found that mutations in key residues at the binding interface not only alter the binding affinity between ziresovir and the F protein but also impair the antiviral efficacy of ziresovir. Collectively, our work clarifies the molecular mechanism underlying the interaction between ziresovir and F protein, and provides a structural basis for the rational design of next-generation anti-RSV inhibitors.

## Introduction

RSV is a leading global cause of acute lower respiratory tract infections (LRTIs) in infants and children under 5 years of age, accounting for a substantial proportion of infant hospitalizations and deaths [1–4]. RSV also causes severe illness in older adults and individuals with chronic and immuno-compromising health conditions. The burden associated with RSV in adults aged 65 years and older is estimated to be comparable to that of seasonal influenza [5–7].

RSV infection initiates in the nasal epithelium and spreads to the lungs, where viral G protein first adheres to host cells and binds to surface receptors. Subsequent fusion of the viral envelope with the host cell membrane releases the viral RNA genome into the cytosol mediated by fusion (F) protein [8–10]. F protein is a type I integral membrane protein which is synthesized as an inactive precursor (F0) and later activated by cleavage of host cell proteases to yield two disulfide-linked subunits, the carboxy-terminal F1 subunit and the amino-terminal F2 subunit [11,12]. Three F1 and F2 monomers assemble to form the mature trimeric F protein. The F protein trimer adopts a metastable prefusion conformation before infection until a triggering event induces its conformational changes to expose the fusion peptide, enable its insertion into the target host cell membrane. This process follows by formation of a six-helix bundle (6-HB) which is hypothesized to drive membrane fusion [13,14]. Viral fusion inhibition is a validated antiviral strategy that has been demonstrated by the approved HIV drug enfuvirtide [15,16].

Despite decades of extensive efforts to develop antivirals for treating RSV infection, the current available treatment option is still supportive care. Aerosolized ribavirin is the first and, to date, the only approved antiviral for RSV treatment, but the drug has limited clinical efficacy, an unfavorable safety profile, and potentially teratogenicity [17,18]. A significant unmet medical need for RSV-specific antivirals persists. Ziresovir, a potent, selective, and orally bioavailable RSV fusion inhibitor, is the only antiviral with positive phase Ⅲ clinical trial results [19]. It is the only antiviral drug that is included in the WHO Paediatric Drug Optimization (PADO) priority list (https://www.who.int/publications/i/item/B09464). In a phase Ⅱ proof-of-concept trial in hospitalized infants with RSV infection, treatment with ziresovir resulted in substantial reduction in viral load and improvement of Wang Bronchiolitis Clinical Score [20]. In a phase Ⅲ pivotal trial, ziresovir treatment confirmed the previously reported clinical efficacy as well as the antiviral activity [21]. Furthermore, a two-year follow-up study showed that treatment with ziresovir could have long-term health benefits by reducing the annual incidence of parent-reported wheezing, number of wheeziing episodes, and asthma [22]. To elucidate the molecular mechanism of action, we previously reported a computational modeling analysis of ziresovir binding to RSV F protein [19]. In this study, we determined the cryo-EM structure of RSV prefusion F (preF) protein in complex with ziresovir at 3.27Å resolution. The cryo-EM structure reveals a single ziresovir molecule bound within the central cavity of preF protein trimer, stabilized by a network of polar contacts and hydrophobic interactions. Mutational analysis combined with binding affinity measurements of RSV preF viral variants, implicates F140 and D486 as critical contributors to ziresovir binding. These ziresovir-resistant mutants do not propagate as efficiently as the wild-type RSV *in vitro*. Our data provide new structural insight into ziresovir-mediated inhibition of RSV fusion that should facilitate the development of next-generation RSV fusion inhibitors.

## Results

### Antiviral activity of ziresovir against RSV clinical isolates

**Fig 1:**
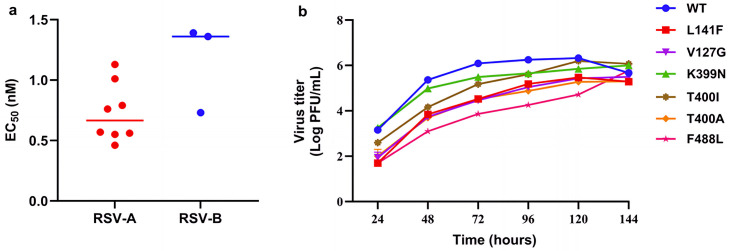
Antiviral activity of ziresovir against clinical isolates and growth kinetics of RSV variants. a, antiviral activity of ziresovir against clinical isolates. The dot represents the EC50 of ziresovir against each RSV clinical isolate. The horizontal line represents the median EC50 against RSV-A or RSV-B. Data are from one representative experiment performed in duplicate. b, comparative growth kinetics of RSV WT and F variants HEp-2 cells were infected with RSV/A2 WT, V127G, L141F, K399N, T400A, T400I or F488L variants at an MOI of 0.1. Viral titers in supernatants at each time point were determined by immunoplaque assay. All values are from one representative experiment performed in duplicate.

Ziresovir demonstrated high potency with EC_50_ values in the single-digit nanomolar level against laboratory strains of RSV [19]. Ziresovir’s antiviral activity was further evaluated against a panel of RSV clinical isolates, including 8 subtype A and 3 subtype B strains. Ziresovir showed comparable activities in the clinical strains compared with laboratory strains of RSV with a median EC_50_ of 0.67 nM (0.46–1.13 nM) and 1.36 nM (0.73–1.39 nM) against RSV-A and RSV-B, respectively (Fig 1a), which suggests that ziresovir has broad activity with a similar efficacy against both RSV A and B strains as well as clinical isolates.

### Viral growth kinetics of recombinant RSV variants

In order to assess the viral fitness of six ziresovir treatment-emergent F protein substitutions (V127G, L141F, K399N, T400A, T400I and F488L) identified in clinical study (ClinicalTrials.gov identifier NCT02654171 and NCT04231968), we generated recombinant RSV variants with residues substitutions in F using reverse genetics approaches based on RSV-A2 sequences. Replication kinetics of wild-type (WT) RSV and F protein variants were evaluated using a cell-based multicycle virus replication assay. All variants exhibited slower replication kinetics than the WT indicating that these residue substitutions reduce replicative fitness of RSV (Fig 1b). At 96 h post-infection, the titer of V127G, L141F, K399N, T400A, T400I and F488L variants were 1.2, 1.1, 0.6, 1.4, 0.6 and 2.0 log_10_ PFU/mL lower than that of the WT, respectively. Nevertheless, K399N and T400I variants were still able to reach peak viral titers comparable to WT.

### Susceptibility to ziresovir of recombinant RSV variants

To determine the impact of ziresovir treatment-emergent residue substitutions on viral susceptibility to ziresovir, the antiviral activity of ziresovir against RSV WT and F protein variants were determined by immuno-fluorescence assay as described above. Three-fold change in EC_50_ between WT and F protein variants was designated as resistance. The V127G substitution in the F protein did not alter susceptibility to ziresovir while the remaining substitutions conferred resistance to ziresovir (Table 1). Among these resistant variants, L141F, T400I, and T400A variants displayed high levels of resistance (>3,703.7-fold) while K399N and F488L variants showed 124.1-fold and 577.4-fold resistance, respectively.

**Table 1:** Susceptibility to ziresovir of RSV F variants containing ziresovir treatment-emergent residue substitutions.

Virus	EC_50_^a^ (nM)	Fold change
**WT**	2.7	1.0^b^
**V127G**	0.8	0.3
**L141F**	>10,000	>3,703.7
**K399N**	335.1	124.1
**T400I**	>10,000	>3703.7
**T400A**	>10,000	>3,703.7
**F488L**	1,559.0	577.4

^a^EC_50_, effective concentration of compound that inhibits 50% of RSV-induced cytopathic effects;

^b^Fold change of the RSV WT was set as 1.0. All values are from one representative experiment performed in duplicate.

### Ziresovir and its derivatives have same binding model predicting through docking

**Fig 2:**
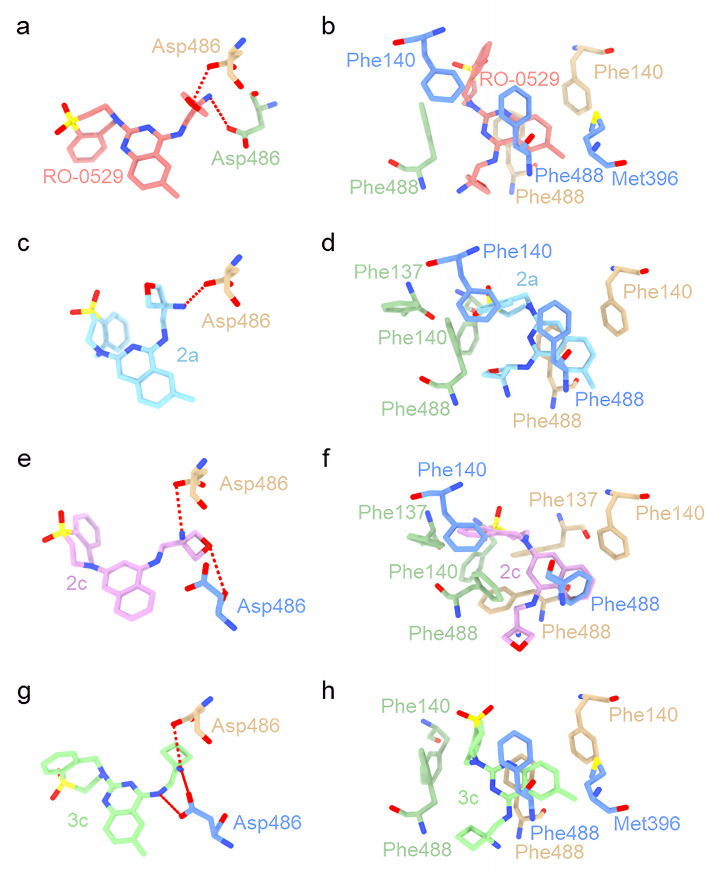
Docking structure of ziresovir (RO-0529) and its derivatives bound to DS-Cav1. The three protomers of DS-Cav1 are depicted in dark sea green, tan, and cornflower blue, respectively, while ziresovir (RO-0529) is illustrated in light coral, with heteroatoms color-coded (nitrogen in blue, oxygen in crimson). Derivatives 2a, 2c, and 3c are rendered in sky blue, plum, light green, respectively. a, c, e and g respectively represent close-up view of the hydrophilic interaction between RO-0529 and its derivatives. b, d, f and h respectively represent close-up shot of the hydrophobic interaction between RO-0529 and its derivatives.

The binding models of ziresovir and its derivatives were investigated through docking studies using a deep learning model, boltz-2, to evaluate their interactions with the RSV preF protein [23]. The docking results indicate that RO-0529 (ziresovir) and its derivatives (2a, 2c, and 3c) can effectively bind to preF protein, with the binding pockets localized within the central cavity of the preF protein [19]. Notably, the binding models for all derivatives exhibit remarkable similarity, with the exception of compound 2c (Fig 2).

The docking scores expressed as pIC_50_ values, are as follows: 7.66 kcal/mol for ziresovir, 7.65 kcal/mol for compound 2a, 7.65 kcal/mol for compound 2c, and 8.64 kcal/mol for compound 3c. Comparison with previously reported docking results achieved using Gold (V5.3), it is observed that Asp486 forms a hydrogen bond with the nitrogen atom of the oxetane side chain in ziresovir and its derivatives (with the exception of 2c) in the boltz-2 docking models. Compound 2c, which features a naphthalene moiety in place of the quinazoline present in ziresovir, exhibits suboptimal binding poses that correspond well with the reported anti-RSV activity. Interestingly, despite possessing a similar docking score to ziresovir, its efficacy appears diminished.

### Structure of fusion glycoprotein in complex with ziresovir

**Fig 3:**
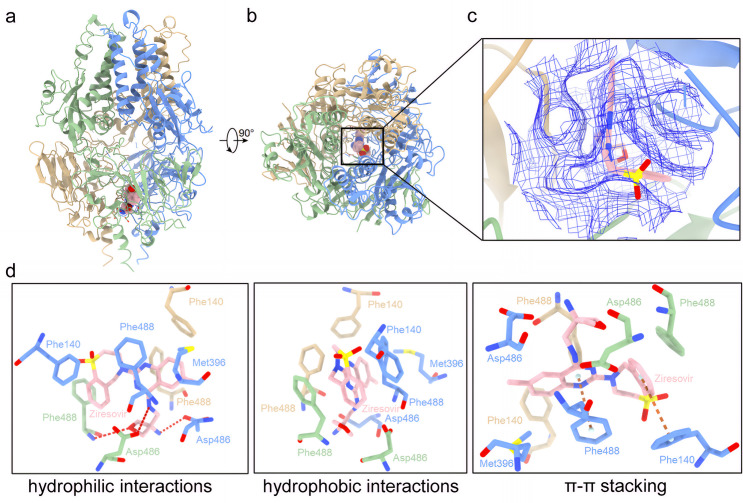
Cryo-EM structure of DS-Cav1 in complex with ziresovir. DS-Cav1 protomers ribbon representation are shown in cornflower blue, dark sea green (denoted with prime symbols), and tan (denoted with double prime symbols), with ziresovir depicted in rose (heteroatoms: N, blue; O, crimson). Coloring conventions are consistent across panels. a, side view of DS-Cav1- ziresovir complex. b, top-down view of ziresovir bound to the DS-Cav1 trimer, illustrating its position at the three-fold symmetry axis within the central pocket. c, a zoom-in view of structural context of ziresovir occupancy in the prefusion F protein trimer (cartoon representation). The preF structure was truncated for optimal visualization of ziresovir, revealing a single copy of ziresovir embedded in the hydrophobic pocket. Cryo-EM density for ziresovir (blue mesh, threshold level at 0.102) highlights its well-defined positioning in the central pocket. d, Close-up views of the binding pocket highlight molecular interactions for ziresovir binding. Left: the hydrogen bonds (red dashed lines) between ziresovir and D486, D486′ (symmetry-related), as well as backbone amide of F488′. Middle: Aromatic stacking involved F488, F488′, F488′′, F140, F140′, F140 and van der Waals interactions with M396 observed for ziresovir hydrophobic packing. Right: specifically, F488 engages in face-to-face π-π stacking with ziresovir’s aromatic core while F140 participates via T-shaped π-π interaction with ziresovir aromatic in benzoazepine moiety.

To elucidate the molecular mechanism of these variants in ziresovir binding to the RSV F protein, we determined a 3.27 Å resolution cryo-EM structure of the RSV preF protein trimer in complex with both motavizumab Fab and ziresovir (Fig 3). Motavizumab Fab purposes to provide additional structural landmarks that facilitated high-resolution reconstruction of this ternary complex without affecting the binding of ziresovir to the F protein, and therefore not included in the figures of this article.

The cryo-EM structure reveals that ziresovir binds within the central cavity of the preF protein, proximal to the fusion peptide (FP) and the heptad repeat B (HRB) region. The reconstructed density map demonstrates that ziresovir occupies a pocket situated along the three-fold rotational axis of the preF trimer. The ligand density itself exhibits three-fold symmetry, with a single ziresovir molecule fitting optimally into the observed density (Fig 3c). Notably, a single ziresovir molecule engages in interactions with all three protomers of the preF protein. Its binding to the F protein is mediated by a combination of hydrophobic and hydrophilic interactions. Specifically, the oxetane group of ziresovir forms polar contacts with the side-chain OD2 of D486, the side-chain OD2 of D486’ and the main-chain nitrogen of F488’. Additionally, extensive hydrophobic interactions occur with F140, M396, F488, and F140’, F488’ (from an adjacent protomer), and F140’‘, F488’‘ (from a third protomer). A critical feature of the binding mechanism is the hydrophobic interactions between the planar aromatic moiety of ziresovir and the surrounding aromatic side chains of F488, F140’, F488’, F488’‘, and F140’‘. F488 engages in face-to-face π-π stacking with ziresovir’s aromatic core while F140 participates via T-shaped π-π interaction with ziresovir aromatic in benzoazepine moiety. These interactions appear to play a pivotal role in stabilizing the preF-ziresovir complex (Fig 3d).

### The variants of F protein that are resistant to fusion inhibitors also correspond to ziresovir interacting residues

**Fig 4:**
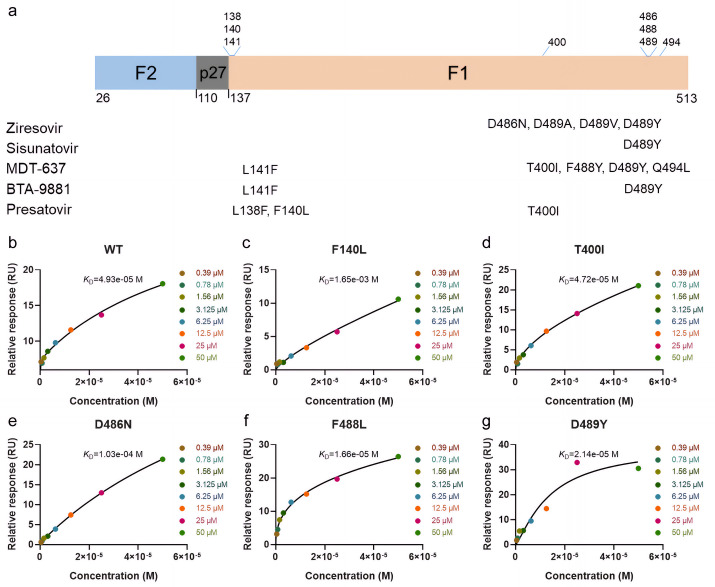
Mutational analysis of RSV F protein and binding affinity characterization. a, domain architecture of the RSV F protein, annotated with resistance-associated sites for fusion inhibitors. b-g, SPR-derived binding kinetics of ziresovir to wild-type DS-Cav1 and resistance variants. Global fitting of dose-response data (solid black curves) demonstrates mutation-dependent affinity changes.

In addition to the mutants mentioned above, other mutants of ziresovir have also been reported [19]. At the same time, most of the inhibitors targeting the RSV F protein exhibit similar effects and produce the similar resistant variant which from clinical resistance and screening for inhibitor resistance *in vitro* (Fig 4a) [24–27]. To investigate the resistance mechanisms, we selected higher frequency mutation site introduced into DS-Cav1 using site-directed mutagenesis, including F140L, T400I, D486N, F488L, D489Y. Subsequently, the variant proteins were purified, and their binding affinities for ziresovir were quantified via surface plasmon resonance (SPR).

DS-Cav1 exhibited an equilibrium dissociation constant (K_D_) of 4.96E-5 M with ziresovir. Under identical conditions, the mutant proteins DS-Cav1-T400I, DS-Cav1-F488L and DS-Cav1-D489Y display K_D_ values of 4.72E-5 M, 1.66E-5 M and 4.93E-5 M, respectively (Fig 4b-4g). Notably, only the F488L substitution resulted in a modest increase in binding affinity. Given that both leucine and phenylalanine are hydrophobic residues, F488 in the preF protein primarily mediates hydrophobic interactions with ziresovir. This observation aligns with structural expectations. Otherwise, DS-Cav1-F140L and DS-Cav1-D486N could not yield reliable K_D_ values in SPR assays, indicating an approximately >10-fold reduction in binding potency for ziresovir. Biochemical evidence from this study underscores the indispensable role of F140 and D486 in mediating ziresovir binding. These findings further corroborate that resistance mutations F140L, D486N, and F488L critically influence the interaction between the F protein and ziresovir. Furthermore, the T400I and D489Y mutations did not directly affect the binding affinity between the preF trimer and ziresovir. This observation is supported by our structural analysis, which revealed that the T400 and D489 are relatively distant from ziresovir’s binding pocket.

## Discussion

Ziresovir represents a groundbreaking innovative medicine in RSV therapeutics as the first anti-RSV drug with positive phase Ⅲ clinical results [21]. With significant effect on improving clinical symptoms and a favorable safety profile, preclinical research indicates that ziresovir possesses broad-spectrum anti-RSV activity and shows high potency against laboratory strains of RSV and a panel of RSV clinical isolates, including both subtype A and subtype B strains [19].

Ziresovir could block the cell fusion induced by RSV F protein in cell-cell fusion experiments. Additionally both drug resistant analysis and in silico docking results further prove that ziresovir acts as a direct RSV F protein inhibitor [19]. Meanwhile, treatment-emergent residue substitutions in F protein by ziresovir were observed during clinical study. These F protein mutations exhibit varying degrees of influence on the replication of RSV. *In vitro* studies confirm that recombinant RSV variants harboring F protein substitutions (L141F, T400I, T400A, K399N, F488L) exhibit strain-specific against ziresovir. These mutations recapitulate a broader resistance signature observed across multiple RSV fusion inhibitors, including MDT-637, BTA-9881. Notably, recurrent substitutions (L141F, D489Y, T400I) implicate a conserved binding pocket for this pharmacophore class of antiviral agents [28–30]. However, the molecular mechanisms underlying the resistance in these variants have not been fully elucidated, warranting further structural and functional studies to elucidate how these mutations disrupt ziresovir binding while preserving viral fitness.

The cryo-EM structure of preF-ziresovir complex presented herein provides critical insights into the molecular mechanism of RSV fusion inhibition. This structure reveals that ziresovir binds to a hydrophobic pocket adjacent to the FP domain of the preF trimer. This binding mode aligns with previous structural studies of other RSV fusion inhibitors and prior docking predictions [19,24,31,32]. The structural evidence provides direct validation of ziresovir’s mechanism as a canonical RSV fusion inhibitor. While numerous studies suggest that fusion inhibitors of this class bind to the preF trimer with a stoichiometry of one inhibitor per trimer, structural analyses to date have been unable to unambiguously confirm this binding ratio due to the inhibitor’s unique positioning along the three-fold symmetry axis. The density in this region remains challenging to resolve at current resolutions, necessitating higher-resolution structural studies to definitively establish the binding stoichiometry.

To elucidate the binding mechanism, we leveraged cryo-EM structural analysis, augmented by Boltz-2, a next-generation biomolecular foundation model capable of concurrent structure prediction and binding affinity estimation. Boltz-2 employs an all-atom co-folding approach, achieving accuracy comparable to physics-based free-energy perturbation (FEP) methods. This model was applied to analyze the binding modes and affinities of RO-0529 (ziresovir) and its derivatives (compounds 2a, 2c, and 3c), which exhibit varying anti-RSV potencies. Docking simulations indicate that compound 2c which substitutes ziresovir’s quinazoline moiety with a naphthalene group also binds to the central cavity of preF protein but adopts a suboptimal binding pose. Comparative analysis of the preF-ziresovir complex structure and docking results suggests that the N35 and N40 atoms of ziresovir’s quinazoline ring are critical for aromatic group of quinazoline interaction with the aromatic side chains of F488 and F140, explaining the diminished antiviral efficacy of compound 2c. During *in silico* docking simulations, three molecules of ziresovir were modeled in complex with the preF trimer. However, structural analysis indicates that the central cavity of the F protein may lack sufficient volume to accommodate three ligands simultaneously. Integrating cryo-EM structural data with docking results, we identified F140, F488, M396 and D486 as key residues mediating interactions between F protein and ziresovir. We also compared the interaction between ziresovir and the RSV F protein predicted by the docking protocol with that observed in the experimental cryo-EM structure, identifying differences between the two models. Specifically, the spatial orientation of ziresovir’s oxetan-3-amine group is inconsistent, while its benzothiazepine moitety exhibits an angular deviation. In experimental structure, the oxetan-3-amine group of ziresovir forms polar contacts with the side-chain OD2 of D486 and the main-chain nitrogen of F488’. Additionally, its secondary amino nitrogen forms polar contacts with the side-chain OD2 of D486’. In contrast, in the docking structure shows that the oxetan-3-amine group of RO-0529 forms polar contacts with the side-chain OD2 of D486 and the side-chain OD2 of D486’‘. Although the result of docking does not exactly match the aforementioned experimental structure we have explained, the position of the oxetan-3-amine group emphasizes the significance of polar interaction at D486 in the preF trimer.

As is well established, it is a critical step for viral fusion with the host cell membrane that the F protein undergoes an irreversible conformational transition from the prefusion to the postfusion (postF) state. This transition involves extensive structural rearrangements, including the refolding of two distinct regions: Refolding Region 1 (RR1, residues 137–216), which harbors the fusion peptide and heptad repeat A (HRA), and Refolding Region 2 (RR2, residues 460–524) which contains HRB. In the postF conformation, HRA and HRB coalesce into a stable 6-HB. Notably, ziresovir interacts with both RR1 and RR2, consistent with its classification as a fusion inhibitor. The preF-ziresovir complex structure further elucidates the molecular interactions that govern inhibitor binding to the preF trimer. Based on the analysis of the ziresovir binding model, we selected the substitutions F140L, T400A, D486N, F488L, and D489Y for SPR assays. Among these, F140L, D486N, and F488L significantly alter ziresovir binding affinity, with a hydrophobic residue (F140) and a polar residue (D486) exerting the most pronounced effects on protein-ligand interactions. The D486N substitution likely disrupts hydrogen bonding and polar interaction networks, whereas F140L preserves hydrophobicity but may perturb the π-π stacking interactions critical for stabilizing the preF conformation. However, the functional impacts of these mutations, as validated by site-directed mutagenesis, does not fully correlate with resistance profiles observed in clinical RSV strains or *in vitro* resistance screening. Notably, F488L exhibits intermediate binding and resistance phenotypes in both SPR and immunofluorescence assays. Intriguingly, T400I mutation does not directly impair the binding affinity between the preF trimer and ziresovir could be explained by T400 is relatively distant from ziresovir’s binding pocket in our cryo-EM structure. Differential scanning fluorimetry (DSF) results demonstrated that the stability of all variant proteins was reduced (S5 Fig). Additionally, the T400I mutant exhibited slower replication kinetics compared to the wild-type (WT) virus, indicating that T400I diminishes the replicative fitness of RSV. These findings lead us to hypothesize that T400I mutation compromises the stability of the preF protein on the virion surface.

Collectively, these findings report the first complex structure of a novel phase III clinical anti-RSV drug (ziresovir) with its target RSV F protein, while experimentally validating the drug’s compatibility with clinical strains and its relationship with potential resistance mutations. Furthermore, this work establishes a structural framework for designing next-generation fusion inhibitors which enhanced efficacy against resistant viral variants.

## Materials and methods

### Cells, viruses, and compounds

HEp-2 cells (ATCC CCL-23) were cultured in Dulbecco modified Eagle medium (DMEM) (Hyclone) with 10% fetal bovine serum (FBS) (Gibco) and 100 U/mL penicillin-streptomycin (Hyclone) at 37°C with 5% CO_2_. BHK21/T7 cells stably expressing the T7 RNA polymerase were maintained in Eagle’s minimum essential medium (EMEM) (ATCC) supplemented with 10% FBS, 100 U/mL penicillin-streptomycin, and 250 μg/mL G418 (Gibco). All clinical isolates of RSV including 8 RSV-A strains and 3 RSV-B strains tested in this study were from Beijing Children’s Hospital. RSV was propagated in HEp-2 cells. RSV fusion inhibitor ziresovir was synthesized in-house and dissolved in dimethyl sulfoxide (DMSO) for antiviral experiments.

### Generation of recombinant RSV strains

To generate the recombinant RSV strains with mutation in F protein, F genes with specific mutations were synthesized and cloned into a bacterial artificial chromosome (BAC) containing the antigenomic cDNA of RSV A2 (GenBank accession number KT992094.1) by Wuxi AppTec. For rescue of recombinant RSV, 600,000 BHK21/T7 cells in 6-well plate were transfected with RSV antigenomic BAC (1.2 μg) together with helper plasmids N (0.6 μg), P (0.3 μg), M2-1 (0.1 μg), and L (0.2 μg). Thereafter, cells were monitored under microscope for cytopathic effect. The supernatant containing recombinant virus was harvested at 4–6 days post-transfection. The viruses were propagated in HEp-2 cells and the sequences of the glycoprotein ORFs were verified by sequencing.

### *In vitro* growth analyses

Subconfluent HEp-2 cells were infected in duplicate with recombinant RSV at a multiplicity of infection (MOI) of 0.1 in T25 flask (Corning Costar) using 150 μL inoculum. After 1 h of infection at 37°C with 5% CO_2_, the inoculum was removed, and the cells were washed twice with DMEM. DMEM supplemented with 2% FBS was then added to each flask, and the flasks were incubated at 37°C with 5% CO_2_ for 6 days. Supernatants were harvested from each flask every 24 h, and RSV in the supernatant was titrated in duplicate by immunoplaque assay on HEp-2 cells as described previously [33].

### Antiviral assay

To test the antiviral activity of ziresovir against RSV, immuno-fluorescence assay was performed. Briefly, 5,000 HEp-2 cells were seeded in 96 well plates (Corning Costar) and cultured at 37°C, 5% CO_2_ overnight. Next day, 5 μL of 3-fold serially diluted ziresovir (diluted in assay medium with a final DMSO concentration of 0.25%) were added to cells. In cell control and virus control wells, 0.25% DMSO alone was added. After 1.5 h of compound addition, 45 μL of diluted virus (2,500 PFU, which corresponds to an MOI of 0.5 based on initial cell plating density of 5,000 cells/well) was added to each well. After 96 h of incubation at 37°C with 5% CO_2_, the cells were fixed with 4% paraformaldehyde (PFA) for 20 min at room temperature, washed twice with PBS, incubated with 0.25% Triton X-100 in PBS for 20 min, washed twice with PBS and incubated with 50 μL of 1:2,000-diluted mouse anti-RSV F monoclonal antibody (Fitzgerald) for 1 h at room temperature. The cells then were washed three times with PBS-T, incubated with 50 μL of 1:4000-diluted Alexa fluor 488 secondary antibody (Invitrogen) for 1 h at room temperature, washed three times with PBS-T, and fluorescence intensity was quantified using Infinite M200 Multiscan Spectrum (Tecan).

### Expression and purification of proteins

Plasmids encoding cDNA for stable preF protein (DS-Cav1) were synthesized (GenScript) and cloned into the pcDNA3.1 expression vector sequentially fused with T4-fibritin trimerization domain, prescission protease, Strep-Tag II and 6 × His tag at the C-terminal [34,35]. A variety of F mutated constructions contain the following substitution in F proteins (F140L, T400I, D486N, F488L, D489Y) obtained using site-directed mutagenesis method. The plasmids were transient transfected into HEK293 cells in suspension at 37°C for 5 days. Motavizumab heavy variable regions with 6 × His tag at the C-terminal simultaneously and light variable regions were synthesized (GenScript) into pcDNA3.4 expression vector transient co-transfected into HEK293 cells [36].

For each batch of protein purification, cell supernatant was centrifugation at 4500rpm (Backman Avanti JXN-26) for 25min. Then, the protein was purified with Ni^2+^-NTA resin (Cytiva) washing with the resuspension buffer (1 × PBS, 50 mM imidazole, pH7.4). Subsequently, the protein was eluted with elution buffer (1 × PBS, 500 mM imidazole, pH7.4). Eluted F protein was concentrated using a centrifugal filter device with a 50-kDa molecular weight cutoff (MWCO) (Millipore) and further purified by Superose 6 Increase 10/300 GL (Cytiva). The eluted motavizumab Fab were concentrated using a centrifugal filter device (10-kDa MWCO, Millipore) and then purified using Superdex 75 Increase 10/300 GL (Cytiva) (S1 Fig). The final proteins were concentrated and stored in 1 × PBS, pH 7.4 at -80°C.

### Surface plasmon resonance assay

The binding capacity between F mutants and ziresovir were monitored using surface plasmon resonance performed with Biacore 8K+ (Cytiva) at 25°C in the LMV-multiple-cycle mode. F protein and variants were immobilized on a Series S Sensor chip CM5 (Cytiva). The immobilization level of DS-Cav1, F140L, T400I, D486N, F488L, D489Y are 12214.9RU, 12122.0RU, 10920.5RU, 13374.5RU, 12284.6RU, 12551.4RU, respectively. The theoretically upper limit of detection level of DS-Cav1, F140L, T400I, D486N, F488L, D489Y are 29.83RU, 29.60RU, 26.67RU, 32.66RU, 30.00RU, 30.65RU, respectively. The running buffer was 1 × PBS (pH 7.4) containing 0.05% Tween-20 (v/v) and 5% DMSO (v/v). For binding tests, ziresovir was prepared at concentrations of 0.39, 0.78, 1.56, 3.125, 6.25, 12.5, 25 and 50 μM in buffer containing 10 mM PBS pH 7.4, 0.05% Tween 20 (v/v), and 5% DMSO (v/v). Ziresovir was analyzed at a flow rate of 30 μL/min for 120s (association phase), followed by a 400s dissociation phase. Regeneration was performed with 50% DMSO injected for 30s to remove bound analyte (S3 Fig). The equilibrium dissociation constants (binding affinity, K_D_) for each pair of interactions were calculated using the Biacore 8 K evaluation software (Cytiva).

### Cryo-EM sample preparation and data collection

The purified DS-Cav1 protein was mixed with ziresovir at a molar ratio of 1:3 and then incubated with motavizumab Fab at 1.2 times the molar amount of DS-Cav1, incubating them together at 4°C for 30 minutes. The final concentration of DS-Cav1 was 0.7 mg/ml. Sample preparation was performed using a Vitrobot Mark IV (Thermo Fisher Scientific). 4 μL sample was loaded to glow-discharged (60s at 15 mA, 38 mbar) grids (Quantifoil Cu 300 mesh, R 1.2/1.3) under the conditions of 100% humidity, 8°C with blotted for 3.5s.

Dataset was acquired on a Thermo Fisher Krios G4 electron microscope (Thermo Fisher Scientific), equipped with a Falcon 4 detector. The energy filter was set to a slit width of 10 eV to eliminate electrons that have undergone inelastic scattering. Data acquisition operations were performed in EPU software, acquiring images at a magnification of 165,000× with a pixel size of 0.729 Å and a total electron dose of 50 e ⁻ /Å².

### Image processing and 3D reconstruction

The dataset processing was all performed using the CryoSPARC software suite [37]. In details, the movies were motion-corrected using Patch motion correction and estimation of the contrast transfer function (CTF) for each micrograph using Patch CTF estimation. Particles were blobbed picked and template picked on 7,189 micrographs and extracted from micrograph using a 360-pixel box size. After multi-cycles of two-dimensional (2D) classification grouped particles images by their projected orientations, total 139,474 particles selected finally to build three initial models in C1 symmetry using Ab-Initio reconstruction. Following of heterogeneous refinement processes, homogeneous refinement, non-uniform refinement and homogeneous refinement using C3 symmetry with symmetry relaxation (maximization) method, a resolution of 3.27 Å cryo-EM density map was got and the map quality was evaluated using gold-standard Fourier Shell Correlation (FSC), with resolution determined by comparing two independently refined half-maps via the FSC = 0.143 criterion [38]. The procedure of dataset processing is shown in S2 Fig.

### Model building, refinement and 3D visualization

The initial atomic model of ziresovir bound to RSV preF-motavizumab Fab complex was built based on the preF-human antibody D25 complex crystal structure (PDB: 4JHW) and preF-motavizumab complex crystal structure (PDB: 4JLR). The preF and motavizumab crystal structure were fitted into the EM density map using UCSF Chimera [39]. The fitted coordinate and map followed by iterative manual fitting adjustment in Coot and real space refinement in PHENIX [40,41]. The 3D molecule structure of ziresovir was generated by elbow in PHENIX suite and placed into the EM density map using Coot. All figures were drawn using UCSF Chimera and UCSF ChimeraX [39, 42]. The data collection and refinement statistics are presented in S1 Table.

### Boltz-2 prediction

Protein-ligand interaction predictions were performed using Boltz-2 implemented on a local high-performance computing node equipped with an NVIDIA A100-SXM4–40GB GPU. The prediction protocol was configured using YAML-formatted input files specifying: (1) the trimeric RSV F protein sequence and (2) the ligand SMILES string. Computational parameters were optimized for affinity prediction of the small molecule binding. The prediction was executed via the command: “boltz predict input.yaml --out_dir results/ --use_msa_server --output_format pdb --accelerator gpu --affinity_mw_correction”. Multiple sequence alignment (MSA) was generated automatically using the default mmseqs2 server to provide evolutionary constraints for the structural prediction [43]. The predicted binding affinity (ΔG, kcal/mol) was converted to a docking score (pIC50) using the following empirical relationship: pIC50= (6 − affinity_pred_value) ×1.364 where affinity_pred_value represents the model-derived binding affinity (IC50) estimate (in μM).

### Differential scanning fluorimetry

The differential scanning fluorimetry (DSF) assay employed a dye-based approach, performed using a BioRad CFX96 real-time fluorescent quantitative PCR instrument with 0.2mL low-profile eight-tube strip, the specific experimental procedures as follows: The 5000 × SYPRO Orange Protein Gel Stain (Sigma Aldrich) was diluted 100-fold to a 50 × SYPRO Orange Protein Gel Stain. Ziresovir dissolved in DMSO was added in a volume of 1 μL at a concentration of 1 mM, incubated with 3 μg purified mutant proteins to a final volume of 27 μL; 3 μL of the 50 × SYPRO Orange Protein Gel Stain was also added and incubated at RT (25°C) for 20 min. The instrument program was set as follows: an initial hold at 25°C for 3 minutes, followed by a temperature increment of 0.5°C per minute within the range of 25°C to 95°C. Data processing was carried out using GraphPad Prism 8. The melting temperature (T_m_), defined as the temperature at which 50% of the protein undergoes denaturation, was determined by analyzing the negative first derivative curve of the temperature function exported by the instrument, and in the obtained curve, the melting temperature corresponds to the lowest point of the curve.

## Supporting information

S1 Fig Purification and analysis of DS-Cav1 and its mutants by size-exclusion chromatography (SEC) and SDS-PAGE. a–f, SEC elution profiles and corresponding Coomassie-stained SDS-PAGE (left) of: a, DS-Cav1, b–f, DS-Cav1 mutants (F140L, T400I, D486N, F488L, D489Y). g, SDS-PAGE analysis of purified motavizumab Fab under reducing conditions. *DS-Cav1-T400I purified using Superdex 200 Increase 10/300 GL.
(TIF)

S2 Fig Cryo-EM structural determination of the RSV F–ziresovir–motavizumab Fab complex. a, schematic workflow of cryo-EM data processing, highlighting key steps in 3D reconstruction. b, angular distribution plot of particle orientations used for final reconstruction. c, Gold-standard Fourier shell correlation (GSFSC) curve for the final refined map (resolution indicated at FSC = 0.143). d- e, local resolution estimation of the final cryo-EM map, displayed in (d) side view and (e) top view (color scale in Å). (TIF)

S3 Fig Binding affinity of ziresovir to wild-type DS-Cav1 and F variants. a-f, biocore sensorgram of SPR Fc 2–1 at concentrations of 0.39, 0.78, 1.56, 3.125, 6.25, 12.5, 25 and 50 μM. (TIF)

S4 Fig The density of preF-ziresovir complex binding pocket. a, local resolution estimation of the binding pocket of the final cryo-EM map (color scale in Å). b, the density within a 5 Å distance around ziresovir. c, the density within a 2.5 Å distance around the residues that have interaction with ziresovir. (TIF)

S5 Fig Differential scanning fluorimetry analysis of F variants with ziresovir. (TIF)

S1 Table Cryo-EM data collection, refinement and validation. (PDF)
