# Supplementary figures and images for: Molecular mechanism of ziresovir targeting the fusion glycoprotein of respiratory syncytial virus
Source: PLoS Pathog. 2026 Jan 23;22(1):e1013864. doi: 10.1371/journal.ppat.1013864 (PMC12863690; doi:10.1371/journal.ppat.1013864)

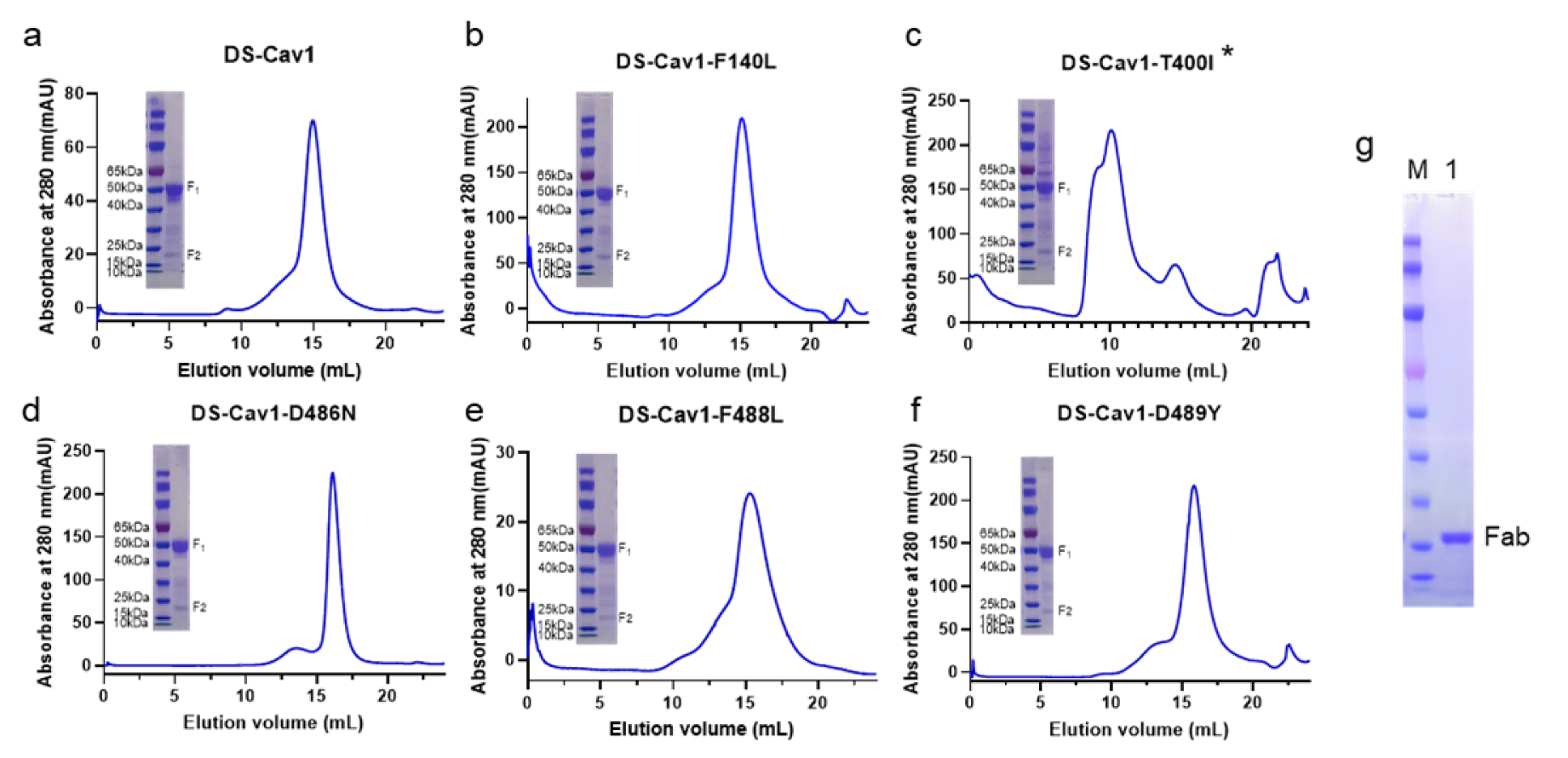

Supplement: S1 Fig — *DS-Cav1-T400I purified using Superdex 200 Increase 10/300 GL. (TIF) [file ppat.1013864.s001.tif]

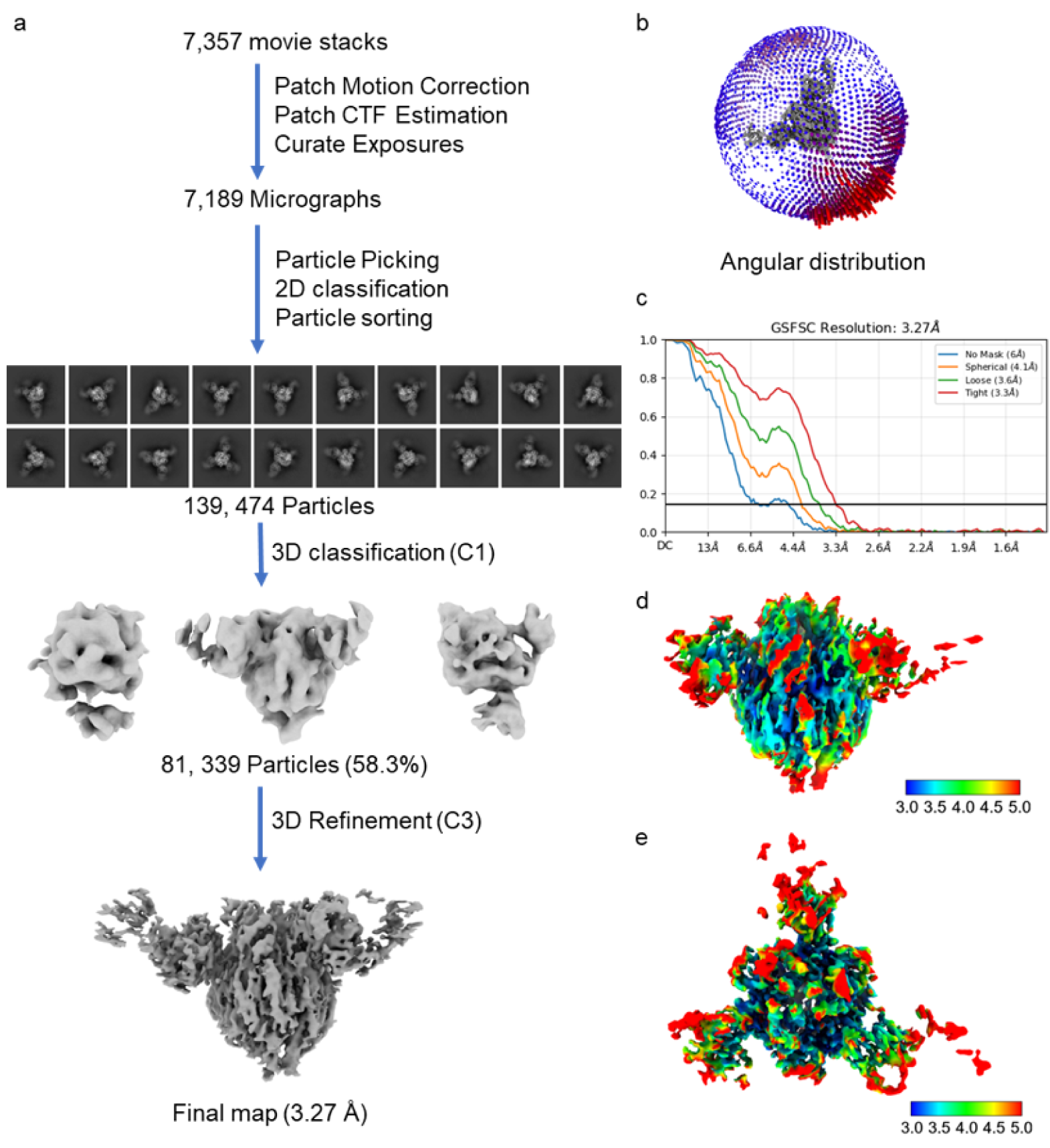

Supplement: S2 Fig — (TIF) [file ppat.1013864.s002.tif]

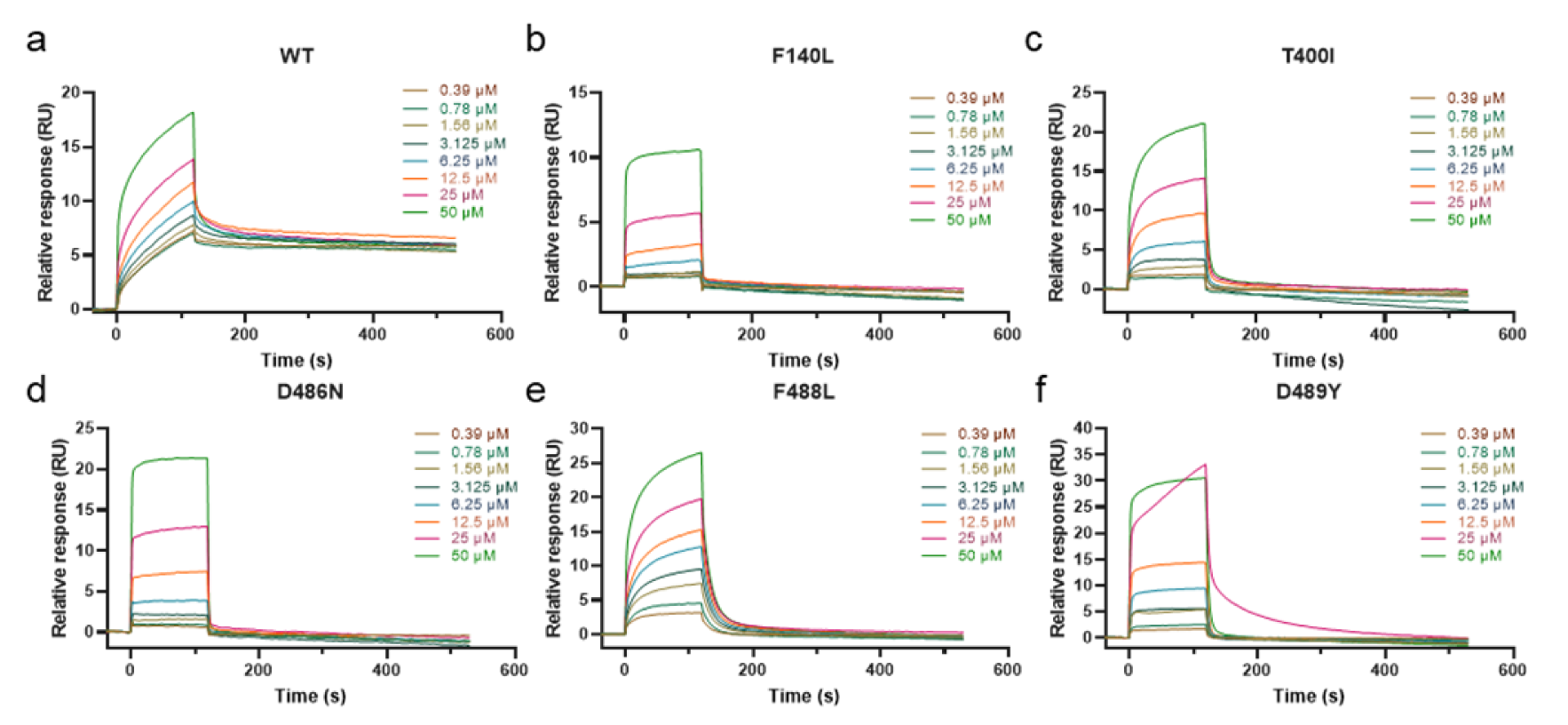

Supplement: S3 Fig — (TIF) [file ppat.1013864.s003.tif]

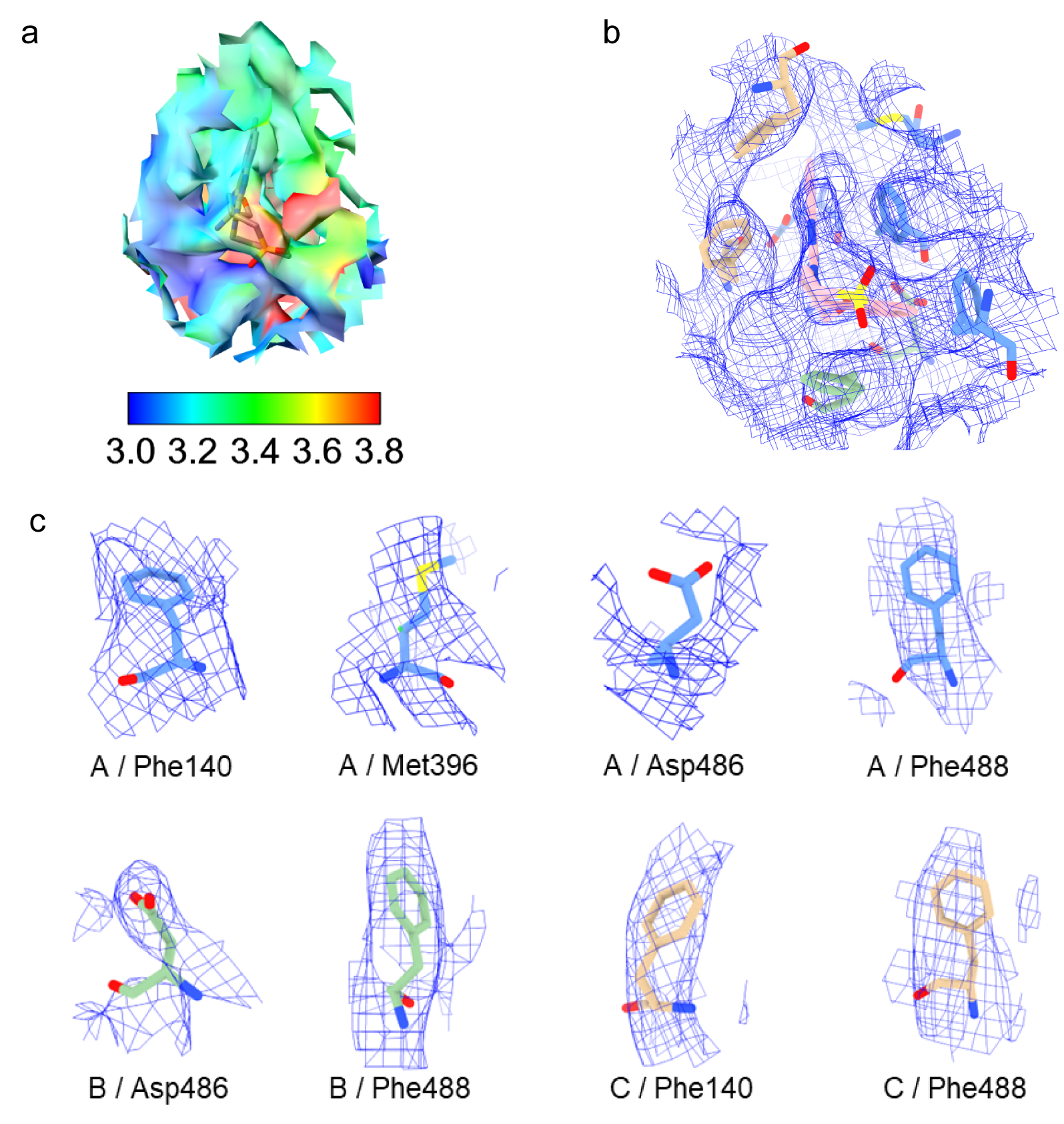

Supplement: S4 Fig — (TIF) [file ppat.1013864.s004.tif]

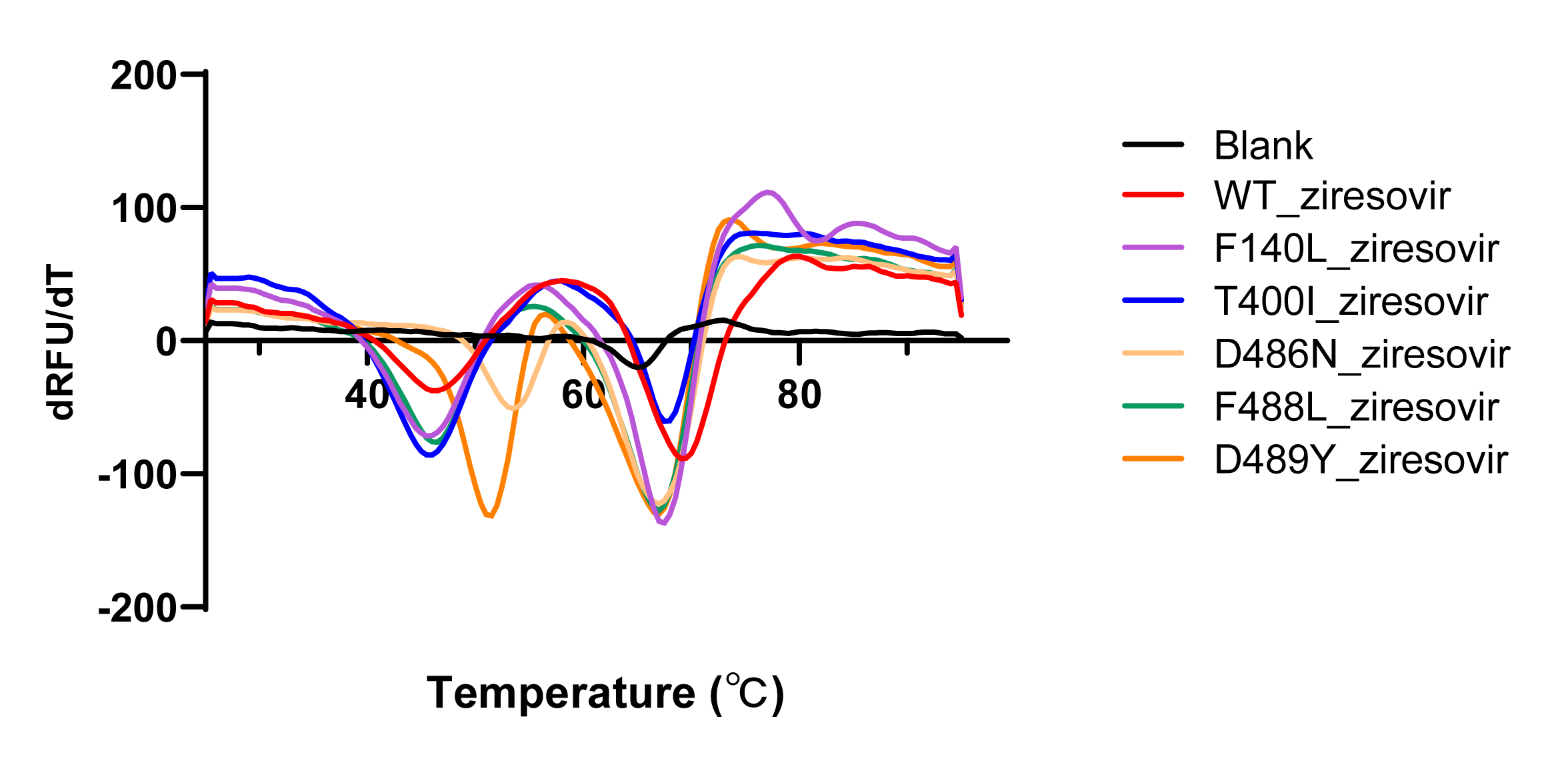

Supplement: S5 Fig — (TIF) [file ppat.1013864.s005.tif]
