# Supplementary material for: Molecular mechanism of ziresovir targeting the fusion glycoprotein of respiratory syncytial virus
Source: PLoS Pathog. 2026 Jan 23;22(1):e1013864. doi: 10.1371/journal.ppat.1013864 (PMC12863690; doi:10.1371/journal.ppat.1013864)
Supplement: S1 Table — (PDF) [file ppat.1013864.s006.pdf]

S1 Table. Cryo-EM data collection, refinement and validation

	DS-Cav1 complex with ziresovir and motavizumab Fab (PDB: 9WDP)
**Data collection and processing**	
Magnification	165,000 ×
Voltage (kV)	300
Electron exposure(e-/Å^2)	50
Defocus range (μm)	-1.2 to -2.4
Pixel size (Å)	0.729
Symmetry imposed	C3
Initial particle images (no.)	139,474
Final particle images (no.)	81,339
Map resolution (Å)	3.27
FSC threshold	0.143
Map resolution range (Å)	250-3.27
**Refinement**	
Initial model used (PDB code)	4JHW, 4JLR
Model resolution (Å)	3.20
FSC threshold	0.5
Model resolution range (Å)	250-3.20
Map sharpening B factor (Å)	-62.4
Model composition	
Non-hydrogen atoms	11338
Protein residues	1396
Ligands	1
B factors (Å)	
Protein	114.29
Ligand	118.99
R.m.s.deviations	
Bond lengths (Å)	0.003
Bond angles (°)	0.651
Validation	
MolProbity score	2.01
Clashscore	14.05
Poor rotamers (%)	0.16
Ramachandran plot	
Favored (%)	94.84
Allowed (%)	5.16
Disallowed (%)	0
